# Efficient Expression of Acetylcholine-Binding Protein from *Aplysia californica* in Bac-to-Bac System

**DOI:** 10.1155/2014/691480

**Published:** 2014-07-20

**Authors:** Bo Lin, Hailing Meng, Hui Bing, Dongting Zhangsun, Sulan Luo

**Affiliations:** Key Laboratory of Tropical Biological Resources, Ministry of Education, Key Lab for Marine Drug of Haikou, Hainan University, Haikou, Hainan 570228, China

## Abstract

The Bac-to-Bac baculovirus expression system can efficiently produce recombinant proteins, but the system may have to be optimized to achieve high-level expression for different candidate proteins. We reported here the efficient expression of acetylcholine-binding proteins from sea hares *Aplysia californica* (Ac-AChBP) and a convenient method to monitor protein expression level in this expression system. Three key factors affecting expression of Ac-AChBP were optimized for maximizing the yield, which included the cell density, volume of the infecting baculovirus inoculums, and the culturing time of postinfection. We have found it to reach a high yield of ∼5 mg/L, which needs 55 h incubation after infection at the cell density of 2 × 10^6^ cells/mL with an inoculum volume ratio of 1 : 100. The optimized expression system in this study was also applied for expressing another protein Ls-AChBP from *Lymnaea stagnalis* successfully. Therefore, this established method is helpful to produce high yields of AChBP proteins for X-ray crystallographic structural and functional studies.

## 1. Introduction

Acetylcholine binding proteins (AChBPs) have been identified from different snails, including* Lymnaea stagnalis* (Ls-AChBP) [1],* Aplysia californica* (Ac-AChBP) [2], and* Bulinus truncatus *(Bt-AChBP) [3]. AChBPs are water-soluble proteins produced and stored in glial cells and released in an acetylcholine-dependent manner into the synaptic cleft, where they modulate synaptic transmission. The mature AChBPs form a stable homopentamer. AChBPs are found to be homologous to the ligand-binding domains of the nicotinic acetylcholine receptors (nAChRs), and they also have similar pharmacological properties [4–6]. Most of conserved residues within the nAChRs family are present in AChBPs, including those relevant to ligand bindings. Moreover, AChBPs bind to the known nAChRs agonists and compete with antagonists such as acetylcholine, nicotine, D-tubocurarine, and *α*-bungarotoxin. Therefore, AChBPs can be used as a model of the ligand-binding domain of nAChRs [7–9].

AChBPs have been previously cocrystallized with prototype ligands that are also known to bind to nAChRs, thus, establishing the structural determinants for a similar ligand recognition of agonists such as nicotine, carbamylcholine [10], and lobeline [11] and antagonists such as different *α*-conotoxins [12, 13] and long-chain snake neurotoxins [14]. Comparison of the ligand bound and unbound crystal structures has revealed the conformational changes of AChBPs or nAChRs upon the ligand binding. These conformational changes may be coupled to the ion channel opening through the loops that form the interface with transmembrane dome in nAChRs [4].

Many *α*-conotoxins have a better affinity for AChBP from* Aplysia californica* (Ac-AChBP) than that from* Lymnaea stagnalis* or* Bulinus truncates*. They have been demonstrated to selectively bind to human *α*7 nAChR or other homomeric nAChRs. The Ac-AChBP has the most resemblance to human *α*7 nAChR in the protein sequence similarity [1, 2, 15]. The alignment of protein sequences of Ac-AChBP with human *α*7 nAChR subunit shows the amino acid sequence identity is 26%, but the sequence similarity is 42.9%, and many residues among them are conserved such as the conserved tryptophan (W) in the ligand binding site [1].

A monomeric Ac-AChBP protein molecule, including its precursor, is about 27 KD in molecular weight, but mature Ac-AChBP (~25 kD) usually forms the pentamer similar to nAChRs. The Ac-AChBP molecule was also reported to be cocrystallized with *α*-conotoxin to elucidate human *α*7 nAChR or other homomeric nAChRs structures and function. It is hopeful for us to use this structural information to design new drugs for pain, addiction, Parkinson's disease, Alzheimer's disease, and epilepsy [16–19].

We have been working on a novel and atypical *α*-conotoxin LtIA from* Conus litteratus* previously discovered from South China Sea [15]. We want to further explore the binding of this atypical *α*-conotoxin LtIA with the receptor Ac-AChBP. Therefore, we reported here a suitable method for efficient expression and purification of Ac-AChBP proteins in the Bac-to-Bac expression system for Ac-AChBP cocrystallized with *α*-conotoxin LtlA to obtain the complex crystal structures.

Efficient expression of Ac-AChBP proteins is an essential step for crystallography research. This study has established a reliable and more convenient method for expressing Ac-AChBPs in a high yield for structural and functional studies. The optimized conditions and the convenient expression level testing method of Ac-AChBP can be also applied to other similar recombinant proteins such as nAChRs.

## 2. Material and Methods

### 2.1. Ac-AChBP Expression in Bac-to-Bac Baculovirus System

Bac-to-Bac baculovirus expression system (Invitrogen) was used to express recombinant Ac-AChBP protein. Sf9 insect cells were maintained in the Insect-Xpress protein-free medium (Lonza) without serum. Ac-AChBP receptor gene from* Aplysia californica* (GenBank: NM_001204559) was used for expression. The synthesized 708 bp (236 aa) of soluble acetylcholine precursor with an N-terminal signal peptide (1–19 aa) and a C-terminal 6×His tag was cloned into pFastBac 1 vector (Invitrogen). The cloned vector was transformed into bacterial DH10Bac competent cells for making recombinant bacmid. The recombinant bacmid was then extracted and transfected into Sf9 cells by Cellfectin II Reagent (Invitrogen). The P1 viruses were harvested after incubation of the transfected cells at 27°C for 7 days and tested, and then they were amplified for two more rounds. The P3 viruses were used to infect 1 L Sf9 cells at a density of 1~4 × 10^6^ cells/mL to express protein [20].

### 2.2. Testing Extracellular (Secreted) Expression of Ac-AChBP of P1 Baculovirus

We tested extracellular expression of Ac-AChBP P1 baculovirus as follows: 20 *μ*L P1 baculovirus was added into 2 mL Sf9 cells (in 6-well plates) and harvested on 48 h after infection. The medium (about 2 mL) mixed with 50 *μ*L nickel- (Ni-) charged resin (GE Healthcare) and 20 mM imidazole in Tris buffer (50 mM Tris, pH 8.0, 50 mM NaCl) and was shaken for 2 h; then the mixture was centrifuged at 3,000 rpm for 5 min. The supernatant was removed. The precipitation of nickel- (Ni-) charged resin was resuspended by 40 *μ*L 300 mM imidazole in Tris buffer and spun again, and the supernatant was taken for SDS-PAGE gel analysis.

The protein sample of Ac-AChBP for reduced SDS-PAGE analysis was dissolved in reduced buffer containing dithiothreitol (DDT) and also boiled for 5 min, but the protein sample for nonreduced SDS-PAGE analysis was dissolved in nonreduced buffer and also not heated.

### 2.3. Purification of Secreted Ac-AChBP

The supernatant of cell culture containing the secreted Ac-AChBP was harvested after infection for 55 h and concentrated and also buffer-exchanged to Tris buffer (50 mM Tris, pH 8.0, 50 mM NaCl). Ac-AChBP was captured by nickel- (Ni-) charged resin (GE Healthcare) and eluted with 300 mM imidazole in Tris buffer (50 mM Tris, pH 8.0, 50 mM NaCl) then further purified by gel filtration chromatography using the Superdex 200 column (GE Healthcare) [20]. The detection wavelength was 280 nm. Elution was performed with a 50 mM NaCl, 50 mM Tris buffer (pH 8.0) at a flow rate of 0.5 mL/min.

### 2.4. Method for Testing Protein Expression Level

Take 2 mL from the postinfected culture and centrifuge the culture at 3,000 rpm for 10 min, collect the supernatant and add 50 *μ*L nickel- (Ni) charged resin (GE Healthcare) in this Tris buffer (50 mM Tris, pH 8.0, 50 mM NaCl, 20 mM imidazole). The mixture of resin and the protein supernatant was shaken for 2 h. Then, centrifuge the mixture at 3,000 rpm for 5 min and remove supernatant. The precipitation of nickel- (Ni-) charged resin was resuspended by 40 *μ*L 300 mM imidazole in Tris buffer and centrifuged again. The supernatant was finally collected and analyzed by SDS-PAGE gel.

### 2.5. Optimization of Ac-AChBP Expression in Bac-to-Bac System

The optimization experiments were designed by comparing the effects of three major factors on the production of Ac-AChBP. They were performed by infecting 1 L cell culture with baculovirus infection and incubating these cells at a constant temperature of 27°C under shaking (110 rpm). The parameters of three factors were varied as detailed in Table 1. Their expression levels were tested by expression level method.

### 2.6. Purification Intracellular Expression of Ac-AChBP

After infection for 55 h, the culturing cells were harvested and centrifuged at 3,000 rpm for 15 min. The cell pellet was resuspended in 40 mL of Tris buffer (50 mM Tris, pH 8.0, 50 mM NaCl), sonicated on ice 15 min with 3 s/9 s intervals, and then centrifuged at 13,000 rpm for 60 min. The supernatant was collected and the supernatant containing Ac-AChBP protein was further purified as described in purification of secreted Ac-AChBP.

### 2.7. Cocrystallization of Ac-AChBP with *α*-Conotoxin LtIA

The Ac-AChBP protein sample was purified and concentrated to about 1 mg/mL in the Tris buffer. Their concentrations were determined by NanoDrop 2000 Spectrophotometer (at 280 nm). The Ac-AChBP absorbance value is 1.5 (obtained by ProtParam tool, Expasy). Ac-AChBP molecular weight is 27000 Daltons. So, the amount of 1 mL Ac-AChBP (1 mg/mL) is calculated as follows:

(1) the  amount  of  AChBP=(1÷1.5×1×10−3)27000=25 n mol.

1 mL Ac-AChBP (1 mg/mL) was mixed with 25 n mol *α*-conotoxin LtIA [15], and the mol ratio was about 1 : 1. Then, the mixture was equilibrated at room temperature for 2 h; after that, it was centrifuged at 4°C, 13,000 rpm for 15 min and purified by gel filtration chromatography.

The purified Ac-AChBP/LtIA complex fraction was collected and concentrated to 20 mg/mL; then, it was taken for crystallization. We applied the crystal robot selected and optimized reservoir solution. The crystals were observed after 3 days under microscopy. The optimized reservoir solution was 0.8 M lithium sulfate monohydrate, containing 0.1 M sodium acetate trihydrate pH 4.6. The generated crystals were applied to X-ray diffraction at the BL17U beam line of the Shanghai Synchrotron Research Facility (SSRF).

## 3. Results and Discussion

### 3.1. Generation of Recombinant Baculovirus and Expression of Ac-AChBP

The synthesized Ac-AChBP gene including precursor (signal peptide) and a C-terminal 6×His-tag was ligated into pFastBac 1 vector. After the confirmation of these fusion peptide sequences and the reading frames by sequencing, this pFastBac 1 vector was transformed into the helper bacterial cells (*E. coli* DH10Bac) for making recombinant bacmids.

**Figure 1:**
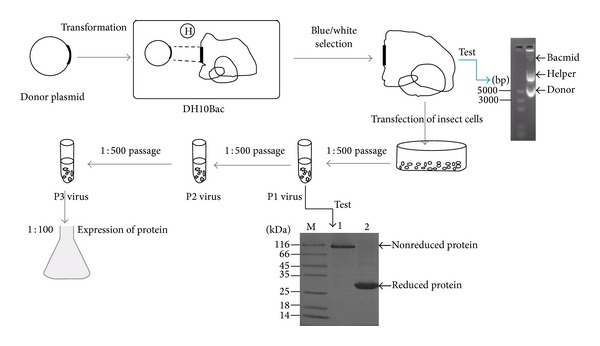
General procedures for expressing Ac-AChBP in the bac-to-bac baculovirus expression system. The Bacmid verification is by agarose gel, but the protein expression test is by SDS-PAGE. P1 indicates passage one, and so on.

As shown in Figure 1, the recombinant bacmid containing Ac-AChBP gene was obtained through transformation into* E. coli* DH10Bac, which was isolated from white colonies and tested by agarose gel electrophoresis (Figure 1, the picture of agarose gel). Then, the bacmid was transfected into insect cells (SF9) to obtain baculovirus passage 1 (P1).

P1 baculovirus protein expression was tested and the result was shown in Figure 1 SDS-PAGE gel. The nonreduced or native protein band was at the top, which indicated the protein formed homopentamer (lane 1). The reduced protein band is about 27 KD, which indicated it was reduced to monomer (lane 2). The P1 baculovirus was amplified at 1 : 500 and cultured for 7 days to obtain P2 baculovirus, and the same way was used to get P3 baculovirus. Finally, we applied P3 baculovirus to express the recombinant protein.

Traditional applied multiplicity of infection (MOI) to infect insect cells needs to know the baculovirus titration such as from the plaque and endpoint dilution assays, which would take 1 to 2 weeks to accomplish. Our method is to test the protein of P1 baculovirus that only needs 48 h. This method would be more straightforward and convenient, and could be used instead of existing titration methods in the bac-to-bac expression system.

### 3.2. Purification of the Secreted Ac-AChBP

**Figure 2:**
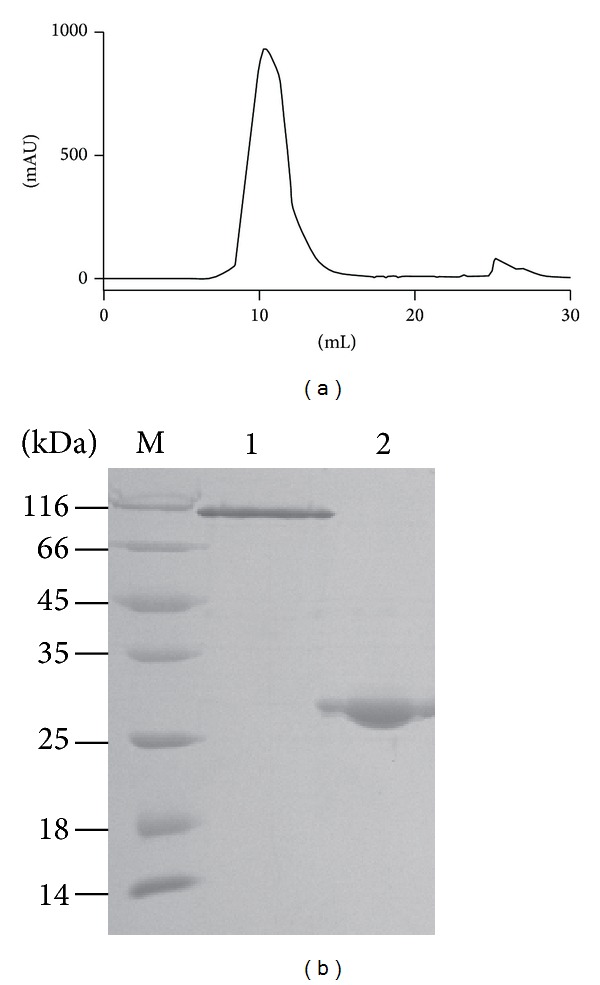
Purification and identification of the secreted Ac-AChBP. (a) Gel filtration chromatography of the Ac-AChBP purification. (b) SDS-PAGE analysis of the Ac-AChBP purification. The nonreduced band was at the top (lane 1), which indicated Ac-AChBP formed pentamer. The reduced band was about 27 KD (lane 2).

The secreted soluble Ac-AChBP was purified by gel filtration chromatography. The eluted chromatography position of Ac-AChBP was in Figure 2(a) at column volume 12.5 mL. The position of 12.5 mL indicated molecular weight of ~13.5 KD according to the instruction of Superdex 200 column (GE Healthcare), proving Ac-AChBP formed pentamer. The small peak at 27 mL indicated imidazole, the following gel filtration chromatography indicated imidazole at the same position.

The Ac-AChBP fractions of gel filtration chromatography were collected for further SDS-PAGE analysis. The nonreduced (native) Ac-AChBP showed as pentamer on the SDS-PAGE gel top (Figure 2(b), lane 1). The reduced Ac-AChBP was ~27 KD which indicated that it was reduced to monomer (Figure 2(b), lane 2).

### 3.3. Optimization of Ac-AChBP Expression

**Figure 3:**
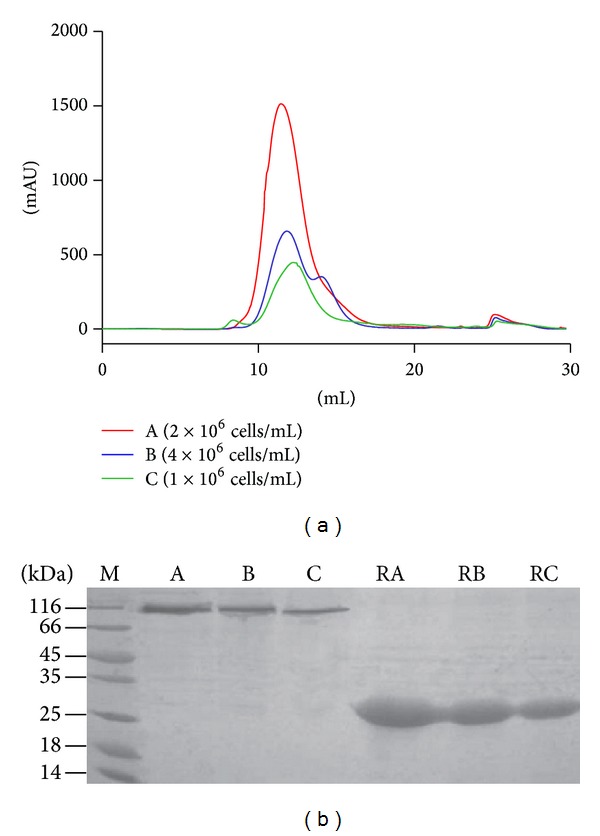
Comparison of two methods used for testing protein expression from different cell densities. (a): Test protein expression level by gel filtration chromatography method different cell densities expression level (from high to low: A, 2 × 10^6^ cells/mL; B, 4 × 10^6^ cells/mL; C, 1 × 10^6^ cells/mL). (b): Test protein expression level by SDS-PAGE method from different cell densities (lane A, 2 × 10^6^ cells/mL; lane B, 4 × 10^6^ cells/mL; lane C, 1 × 10^6^ cells/mL. RA, RB, and RC indicated reduced stated of Ac-AChBP expression samples, respectively, from lanes A, B, and C), the result of test expression level method analysis was the same as gel filtration chromatography.

For expression of Ac-AChBP, we found it was obvious that different conditions had different expression levels, which included the bacmid virus titers, volumes of inoculums, and the postinfection time.  Figure 3(a) shows different cell densities (1 × 10^6^, 2 × 10^6^, 4 × 10^6^ cells/mL) for different Ac-AChBP protein expression levels which were analyzed by gel filtration chromatography. The results indicated that 2 × 10^6^ cells/mL was the highest protein expression level.

Because gel filtration chromatography for testing protein expression level needs to purify 1 L postinfection culture, we found a convenient method to monitor the expression level by SDS-PAGE (as described in Section 2.5). This method only uses 2 mL postinfection culture and the result as shown in the SDS-PAGE gel (Figure 3(b)). The result showed that the sample with the density of 2 × 10^6^ cells/mL had the highest expression level, the same result as tested by gel filtration chromatography method. Because this SDS-PAGE method is only using 2 mL of postinfection culture that does not affect the culture for further expression process, we have used this method for the test in the following optimized experiment to get high yields of Ac-AChBP proteins.

**Figure 4:**
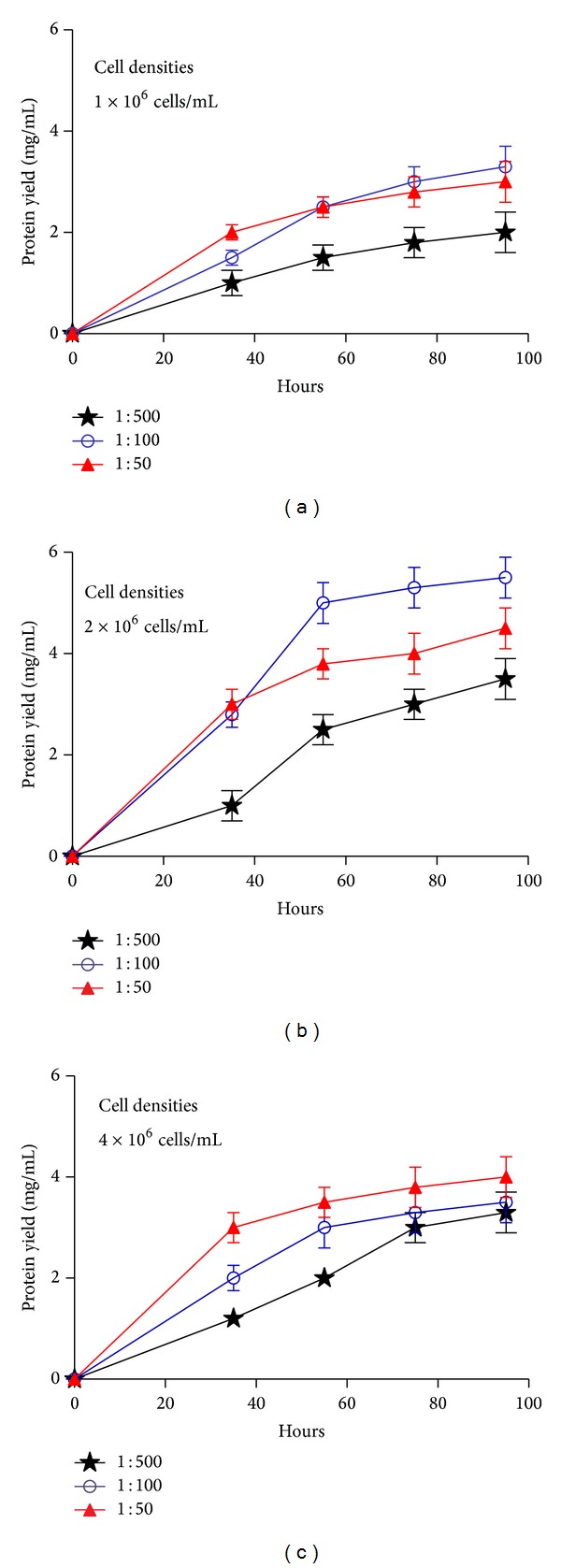
Optimization of Ac-AChBP expression. (a) Cell densities 1 × 10^6^ cells/mL, at different volume of the infecting baculovirus inoculums (1 : 50, 1 : 100, and 1 : 500) were monitored at different time (35 h, 55 h, 75 h, and 95 h). (b) Cell densities 2 × 10^6^ cells/mL, at different volume of the infecting baculovirus inoculums were monitored at different time. (c) Cell densities 4 × 10^6^ cells/mL, at different volume of the infecting baculovirus inoculums were monitored at different time points.

To optimize the yield of Ac-AChBP protein, the parameters in Table 1 were evaluated systematically under the same virus titers (2 × 10^7^ pfu/mL). Sf-9 cells were grown to log phase in 1 L shaking flasks. Infection was carried out at different cell densities (1 × 10^6^, 2 × 10^6^, 4 × 10^6^ cells/mL). Volume of the infecting baculovirus inoculums (1 : 50, 1 : 100, 1 : 500) and Ac-AChBP production were monitored at different postinfection time (35 h, 55 h, 75 h, and 95 h). The results were shown in Figure 4. The Ac-AChBP protein expression reached the highest level of about 5.5 mg/L with a cell density of 2 × 10^6^ cells/mL, harvested at 95 h postinfection, at 1 : 100 volume of the infecting baculovirus inoculums (Figure 4(b), blue line).

**Figure 5:**
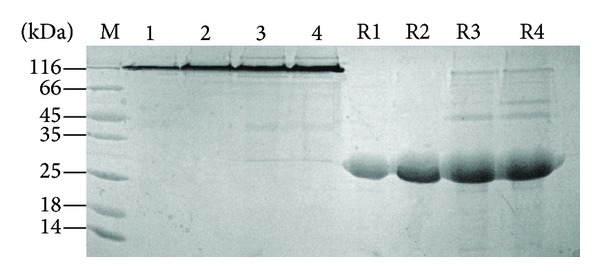
Test expression level method analysis different time expression level postinfection. Expression Ac-AChBP level from high to low, 35 h (lane 1); 55 h (lane 2); 75 h (lane 3); 95 h (lane 4). R1, R2, R3, and R4 indicated reduced stated of Ac-AChBP from lanes 1, 2, 3, and 4.

The protein expression levels were evaluated by the SDS-PAGE gels (Figure 5); it also showed that when the postinfection time was more than 55 h, the cells were dying (lane 3 and lane 4), and the quantity of recombinant protein expression also increased very slowly. So considering pure and high yield of Ac-AChBP protein sample, the best harvest time was 55 h of postinfection, and it can get about 5 mg/L of Ac-AChBP protein under the optimized conditions.

In the optimization experiments, volume of the infecting baculovirus inoculums is corresponding to the cell density. The baculovirus can be amplified together with the cell growth, which is normal for protein expression. However, if the baculovirus is amplified too quickly, the cells will be dying and unhealthy which is not good for protein expression. If the baculovirus is amplified too slowly, the cells will have not been infected efficiently and will affect the expression of the recombinant protein [21].

**Figure 6:**
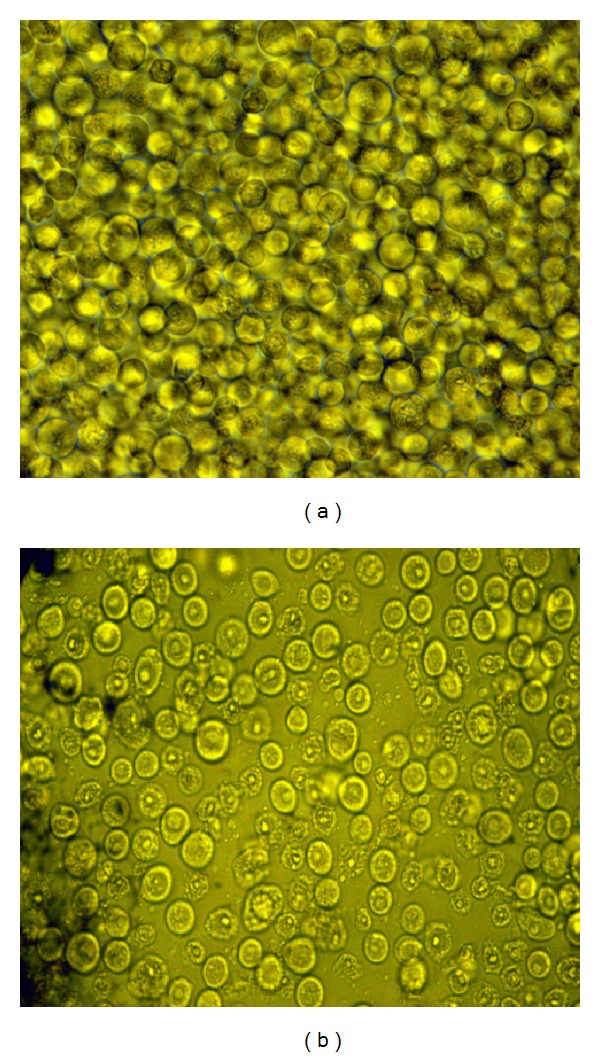
Different Sf9 cells morphology with different post infection time. (a): Sf9 cells with postinfection time before 55 h; (b): Sf9 cells with postinfection time after 55 h, the cells unhealthy and dying.

The best harvest time depended on the time of baculovirus amplified, recombinant protein expression and the transportation from intracellular to extracellular. If the recombinant protein needs more time to be modified and transported to extracellular, it needs more postinfection time. But the postinfection time could not be longer than 55 h, because the cells will be lysed, and it will also affect the protein quality. Thus, the best time for Ac-AChBP expression in this study was 55 h (Figure 6).

### 3.4. Comparison of Ac-AChBP Intracellular and Extracellular (Secreted) Expression

**Figure 7:**
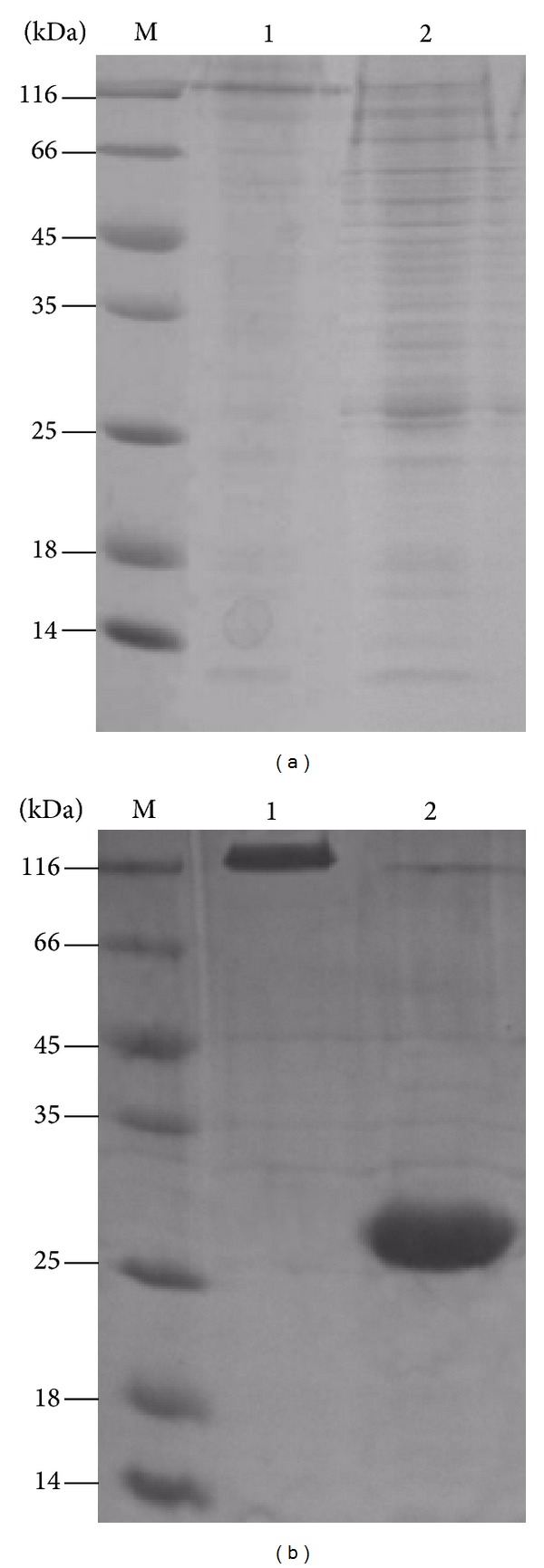
SDS-PAGE analysis of Ac-AChBP intracellular and extracellular expression, (a): Ac-AChBP intracellular expression. Lane 1 nativeAc-AChBP, lane 2 reduced Ac-AChBP, lane M protein marker. (b): Ac-AChBP expression in extracellular. Lane 1 nativeAc-AChBP, lane 2 reduced Ac-AChBP, and lane M, protein marker.

**Figure 8:**
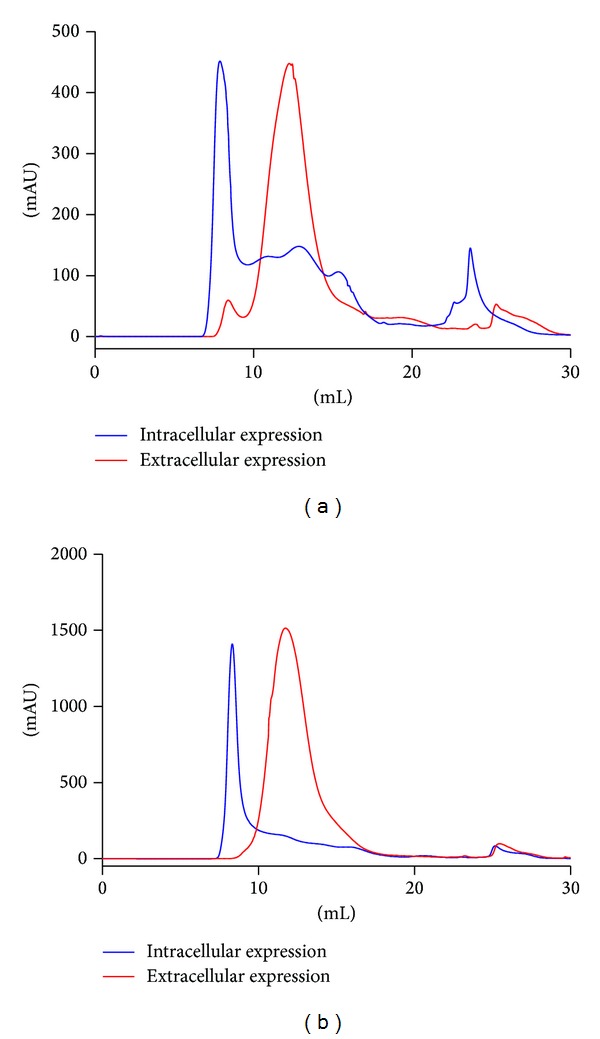
Gel filtration chromatography analysis correlation of Ac-AChBP intracellular and extracellular expression. (a): about 500 mAU peak value expression, the blue line was intracellular expression and the red line was extracellular expression. (b): about 1500 mAU peak value expression, the blue line was intracellular expression and the red line was extracellular expression.

The Ac-AChBP intracellular and extracellular expression was analyzed by SDS-PAGE gel (Figure 7). Although intracellular expressed Ac-AChBP showed as the pentamer, the band was faint. So the intracellular expression was not pure and the expression level was also lower than extracellular. The Ac-AChBP expression in intracellular and extracellular was also analyzed by gel filtration chromatography (Figure 8). It shows that the intracellular and extracellular peak value was correlative; when the intracellular peak value was 500 mAU, the extracellular peak was about 500 mAU too (equivalent to 1.7 mg/L expression level); when the intracellular peak value was 1500 mAU, the extracellular peak was also about 1500 mAU (equivalent to 5 mg/L expression level). It could be concluded that the intracellular expression manipulated extracellular expression or extracellular expression depended on baculovirus amplification. After 55 h of postinfection, the cells were unhealthy and dying, so the extracellular expression was slowed down.

The optimized expression and purification of Ac-AChBP in Bac-to-Bac baculovirus system had also applied for expression of another similar protein Ls-AChBP (GeneBank number AF364899.1) and achieved very good results similar to those of Ac-AChBP (data not shown). However, the Ls-AChBP had higher expression level than Ac-AChBP. The best culturing time for Ls-AChBP expression was 48 h and about 5.5 mg/L purity protein was achieved. Thus, it is apparent that different protein expressions will need different conditions for achieving the best production rate.

### 3.5. Cocrystallization of Ac-AChBP with *α*-Conotoxin LtIA

**Figure 9:**
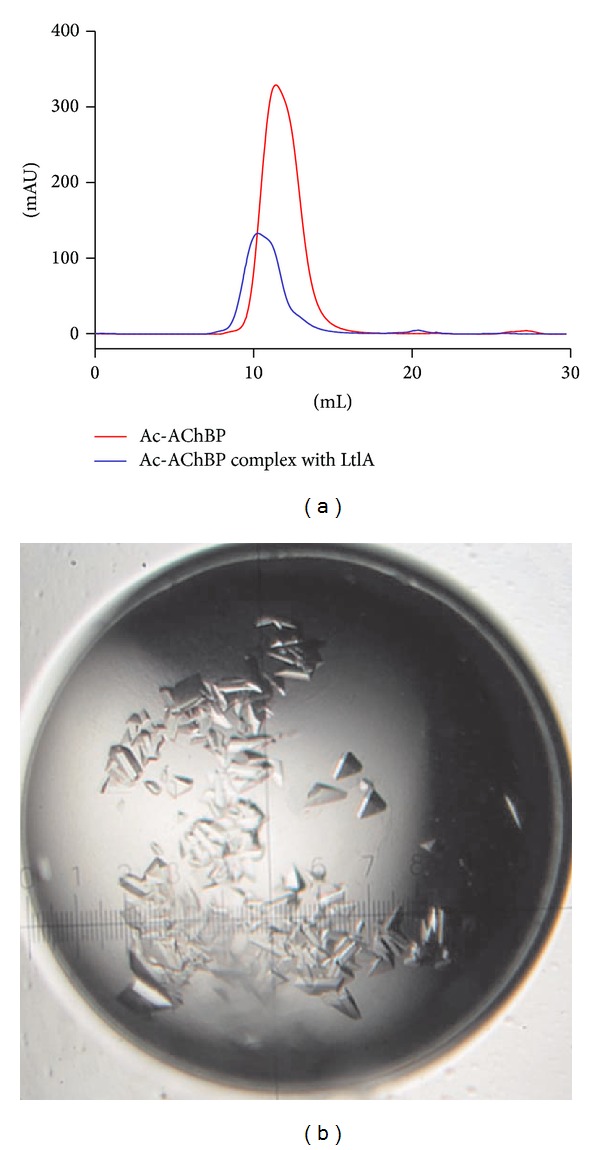
Gel filtration chromatography and crystals of Ac-AChBP complex with *α*-conotoxin LtIA. (a) Comparison of Ac-AChBP (red line) and Ac-AChBP/LtIA (blue line) complex by gel filtration chromatography. (b) The crystals of co-crystallized receptor Ac-AChBP with its ligand *α*-conotoxin LtIA from* Conus litteratus.*

The gel filtration chromatography clearly showed the difference of binding and nonbinding of the ligand of *α*-conotoxin LtIA to the Ac-AChBP receptor (Figure 9(a)). Comparing Ac-AChBP (red line) to Ac-AChBP/LtIA (blue line) complex, both the peak values and the positions are changed by the chromatography. The Ac-AChBP/LtIA (blue line) complex showed molecular weight larger than Ac-AChBP which indicated its binding with *α*-conotoxin LtIA.

The Ac-AChBP/LtIA complex crystals generated from this method were demonstrated to have given high-quality diffraction data (Figure 9(b)). The crystal structures of Ac-AChBP/LtIA complex from these diffraction data will be reported soon at resolution of 2.4 Å. In addition, this method has also helped in protein preparation in solving the structure of Ac-AChBP complex with *α*-conotoxin LvIA [22].

We have applied the efficient expression of Ac-AChBP method to obtain the new crystals of Ac-AChBP complex with *α*-conotoxin LtIA. Many factors will affect protein crystallization, but the key factor is the protein itself. It is essential to get high quality and purified protein from the efficient expression of Ac-AChBP system. We did find that the low level expression of Ac-AChBP could be not easy to get the crystals, it can be explained that low level expression of Ac-AChBP has more aggregation and glycosylation than the efficient expression. This further proved that the efficient expression of Ac-AChBP is critical for getting the high quality crystals of Ac-AChBP complex with the ligand of *α*-conotoxins.

## 4. Conclusions

We have found a convenience method to examine protein expression level and an approach to efficient expression Ac-AChBP in Bac-to-Bac system. We have also found the Ac-AChBP expression behaviors in the intracellular and extracellular compartments of the insect cells. After systematical optimization of the key factors affecting expression of Ac-AChBP, the high yield of ~5 mg/L of Ac-AChBP could be obtained when cell density was 2 × 10^6^ cells/mL, volume of the infecting baculovirus inoculums was in a ratio of 1 : 100, and the cells were harvested at 55 h postinfection. We applied efficient expression of Ac-AChBP to obtain the crystals of Ac-AChBPs complex with *α*-conotoxins. Efficient expression of AChBP is important for protein crystallization and particularly for cocrystallization of AChBP with its ligand *α*-conotoxins. This will contribute significantly to the research on structural and functional studies of AChBPs and nAChRs and the research on *α*-conotoxin-based therapies.

## Figures and Tables

**Table 1:** The three factors and corresponding parameters tested in the optimization experiment.

Factors	Cell density	Volume of infecting baculovirus inoculum	Time of postinfection
Parameters	1 × 10^6^ cells/mL	1 : 50	35 h
	2 × 10^6^ cells/mL	1 : 100	55 h
	4 × 10^6^ cells/mL	1 : 500	75 h
			95 h

## Abbreviations

AChBP: Acetylcholine binding protein
Ac-AChBP: AChBP from* Aplysia californica*
Ls-AChBP: AChBP from* Lymnaea stagnalis*
Bt-AChBP: AChBP from* Bulinus truncatus*
nAChRs: Nicotinic acetylcholine receptors
SDS-PAGE: Sodium dodecyl sulfate-polyacrylamide gel electrophoresis
DTT: Dithiothreitol
MOI: Multiplicity of infection
IPTG: Isopropyl-*β*-D-1-thiogalactopyranoside
RA, RB, and RC: Reduced A, B, and C protein fractions.

## References

[1] Smit AB, Syed NI, Schaap D (2001). A glia-derived acetylcholine-binding protein that modulates synaptic transmission. *Nature*.

[2] Hansen SB, Talley TT, Radić Z, Taylor P (2004). Structural and ligand recognition characteristics of an acetylcholine-binding protein from Aplysia californica. *Journal of Biological Chemistry*.

[3] Celie PHN, Klaassen RV, van Rossum-Fikkert SE (2005). Crystal structure of acetylcholine-binding protein from Bulinus truncatus reveals the conserved structural scaffold and sites of variation in nicotinic acetylcholine receptors. *Journal of Biological Chemistry*.

[4] Dutertre S, Ulens C, Büttner R (2007). AChBP-targeted *α*-conotoxin correlates distinct binding orientations with nAChR subtype selectivity. *EMBO Journal*.

[5] Billen B, Spurny R, Brams M (2012). Molecular actions of smoking cessation drugs at *α*4a*β*2 nicotinic receptors defined in crystal structures of a homologous binding protein. *Proceedings of the National Academy of Sciences of the United States of America*.

[6] Buchapudi K, Xu X, Ataian Y, Ji H, Schulte M (2012). Micromechanical measurement of AChBP binding for label-free drug discovery. *Analyst*.

[7] Kombo DC, Mazurov A, Tallapragada K (2011). Docking studies of benzylidene anabaseine interactions with *α*7 nicotinic acetylcholine receptor (nAChR) and acetylcholine binding proteins (AChBPs): application to the design of related *α*7 selective ligands. *European Journal of Medicinal Chemistry*.

[8] Taylor P, Talley TT, Radic' Z, Hansen SB, Hibbs RE, Shi J (2007). Structure-guided drug design: Conferring selectivity among neuronal nicotinic receptor and acetylcholine-binding protein subtypes. *Biochemical Pharmacology*.

[9] Bourne Y, Radić Z, Aráoz R (2010). Structural determinants in phycotoxins and AChBP conferring high affinity binding and nicotinic AChR antagonism. *Proceedings of the National Academy of Sciences of the United States of America*.

[10] Celie PHN, van Rossum-Fikkert SE, van Dijk WJ, Brejc K, Smit AB, Sixma TK (2004). Nicotine and carbamylcholine binding to nicotinic acetylcholine receptors as studied in AChBP crystal structures. *Neuron*.

[11] Hansen SB, Sulzenbacher G, Huxford T, Marchot P, Taylor P, Bourne Y (2005). Structures of *Aplysia* AChBP complexes with nicotinic agonists and antagonists reveal distinctive binding interfaces and conformations. *The EMBO Journal*.

[12] Celie PHN, Kasheverov IE, Mordvintsev DY (2005). Crystal structure of nicotinic acetylcholine receptor homolog AChBP in complex with an *α*-conotoxin PnIA variant. *Nature Structural and Molecular Biology*.

[13] Ulens C, Hogg RC, Celie PH (2006). Structural determinants of selective *α*-conotoxin binding to a nicotinic acetylcholine receptor homolog AChBP. *Proceedings of the National Academy of Sciences of the United States of America*.

[14] Bourne Y, Talley TT, Hansen SB, Taylor P, Marchot P (2005). Crystal structure of a Cbtx-AChBP complex reveals essential interactions between snake *α*-neurotoxins and nicotinic receptors. *EMBO Journal*.

[15] Luo S, Akondi KB, Zhangsun D (2010). Atypical *α*-conotoxin LtIA from Conus litteratus targets a novel microsite of the *α*3*β*2 nicotinic receptor. *Journal of Biological Chemistry*.

[16] Hansen SB, Radi Z, Talley TT (2002). Tryptophan fluorescence reveals conformational changes in the acetylcholine binding protein. *Journal of Biological Chemistry*.

[17] Azam L, McIntosh JM (2009). Alpha-conotoxins as pharmacological probes of nicotinic acetylcholine receptors. *Acta Pharmacologica Sinica*.

[18] Haeger S, Kuzmin D, Detro-Dassen S (2010). An intramembrane aromatic network determines pentameric assembly of Cys-loop receptors. *Nature Structural & Molecular Biology*.

[19] Arias HR, Blanton MP (2000). *α*-Conotoxins. *The International Journal of Biochemistry and Cell Biology*.

[20] Li L, Wang D, Jiang Y (2011). Crystal structure of human ISG15 protein in complex with influenza B virus NS1B. *Journal of Biological Chemistry*.

[21] Neutra R, Levi BZ, Shoham Y (1992). Optimization of protein-production by the baculovirus expression vector system in shake flasks. *Applied Microbiology and Biotechnology*.

[22] Luo S, Zhangsun D, Schroeder CI (2014). A novel *α*4/7-conotoxin LvIA from Conus lividus that selectively blocks *α*3*β*2 vs. *α*6/*α*3*β*2*β*3 nicotinic acetylcholine receptors. *The FASEB Journal*.

